# Feasibility and safety of planned early discharge following laparotomy in gynecologic oncology with enhanced recovery protocol including opioid-sparing anesthesia

**DOI:** 10.3389/fsurg.2023.1279907

**Published:** 2023-11-03

**Authors:** Michelle L. Kuznicki, Maya Yasukawa, Adrianne R. Mallen, Clarissa Lam, Erica Eggers, Jefferson Regis, Ali Wells, Sarah L. Todd, Sharon E. Robertson, Jean-Paul Tanner, Matthew L. Anderson, Thomas J. Rutherford

**Affiliations:** ^1^ Division of Obstetrics & Gynecology, University of South Florida Morsani College of Medicine, Tampa, FL, United States; ^2^ Women’s Health Institute, The Cleveland Clinic Foundation, Cleveland, OH, United States; ^3^ Department of Gynecology Oncology, Park Nicollet Methodist Hospital, Saint Louis Park, MN, United States; ^4^ Gynecology Service, Department of Surgery, Memorial Sloan Kettering Center, New York, NY, United States; ^5^ Department of Urogynecology, Cooper University Health Care, Camden, NJ, United States; ^6^ School of Medicine, St. George’s University School of Medicine, Great River, NY, United States; ^7^ Department of Internal Medicine, University of Florida, Gainesville, FL, United States; ^8^ Division of Gynecologic Oncology, University of Louisville School of Medicine, Louisville, KY, United States; ^9^ Division of Gynecologic Oncology, Indiana University School of Medicine, Indianapolis, IN, United States; ^10^ Department of Community and Family Health, University of South Florida College of Public Health, Tampa, FL, United States

**Keywords:** ERAS, planned early discharge, quality of life, opioid-sparing anesthesia, gynecology oncology

## Abstract

**Objective:**

This study aims to evaluate the feasibility and safety of planned postoperative day 1 discharge (PPOD1) among patients who undergo laparotomy (XL) in the department of gynecology oncology utilizing a modified enhanced recovery after surgery (ERAS) protocol including opioid-sparing anesthesia (OSA) and defined discharge criteria.

**Methods:**

Patients undergoing XL and minimally invasive surgery (MIS) were enrolled in this prospective, observational cohort study after the departmental implementation of a modified ERAS protocol. The primary outcome was quality of life (QoL) using SF36, PROMIS GI, and ICIQ-FLUTS at baseline and 2- and 6-week postoperative visits. Statistical significance was assessed using the two-tailed Student's *t*-test and non-parametric Mann–Whitney two-sample test.

**Results:**

Of the 141 subjects, no significant demographic differences were observed between the XL group and the MIS group. The majority of subjects, 84.7% (61), in the XL group had gynecologic malignancy [vs. MIS group; 21 (29.2%), *p* < 0.001]. All patients tolerated OSA. The XL group required higher intraoperative opioids [7.1 ± 9.2 morphine milligram equivalents (MME) vs. 3.9 ± 6.9 MME, *p* = 0.02] and longer surgical time (114.2 ± 41 min vs. 96.8 ± 32.1 min, *p* = 0.006). No significant difference was noted in the opioid requirements at the immediate postoperative phase and the rest of the postoperative day (POD) 0 or POD 1. In the XL group, 69 patients (73.6%) were successfully discharged home on POD1. There was no increase in the PROMIS score at 2 and 6 weeks compared to the preoperative phase. The readmission rates within 30 days after surgery (XL 4.2% vs. MIS 1.4%, *p* = 0.62), rates of surgical site infection (XL 0% vs. MIS 2.8%, *p* = 0.24), and mean number of post-discharge phone calls (0 vs. 0, *p* = 0.41) were comparable between the two groups. Although QoL scores were significantly lower than baseline in four of the nine QoL domains at 2 weeks post-laparotomy, all except physical health recovered by the 6-week time point.

**Conclusions:**

PPOD1 is a safe and feasible strategy for XL performed in the gynecologic oncology department. PPOD1 did not increase opioid requirements, readmission rates compared to MIS, and patient-reported constipation and nausea/vomiting compared to the preoperative phase.

## Introduction

The enhanced recovery after surgery (ERAS) program is designed to optimize postoperative recovery (1). Although studies demonstrated that implementing ERAS in gynecologic surgery decreases the postoperative length of stay (LOS) among patients who undergo laparotomy (XL), the mean LOS of patients who undergo gynecologic XL with the ERAS program remains at a range from 3 to 4 days, which is not a substantial improvement in clinical practice (1–3). The limited impact observed could be due to the absence of a universally accepted set of discharge criteria, especially the definition of return of bowel function (4). A systematic review focusing on discharge criteria after colorectal surgery reported that the most cited requirements were tolerance of oral intake (81%) and return of bowel function (70%), with 83% of these criteria specifying the passage of flatus and/or stool. There are currently limited data on definitive endpoints for the return of bowel function, yet this affects the timing of discharge. Emmanuel et al. (4) demonstrated that redefining the return of bowel function, independent of the presence of flatus or fecal passage, allowed for the safe early discharge (within 72 h post-surgery) of patients undergoing colorectal cancer surgery, without increasing the rate of complications. We hypothesized that we could safely discharge patients earlier than previously reported by adopting this endpoint in the field of gynecology.

Pain management is another factor that might contribute to the delay of discharge. Current national guidelines recommend the intraoperative use of short-acting anesthetic agents, such as remifentanil, to facilitate rapid awakening. However, the use of perioperative opioids can impede recovery due to side effects such as sedation, reduced gastrointestinal (GI) motility, nausea, and urinary retention (1). Opioid-sparing anesthesia (OSA) is an alternative approach utilizing multiple, synergistic, non-opioid pharmacologic agents to achieve hypnosis, immobility, sympatholytic, autonomic stability, and analgesia. A previous study reported that OSA has been associated with a reduction in opioid administration both intraoperative phase and during the initial 24 h following surgery without an exacerbation of pain scores, but data are still limited (5).

We aim to evaluate the feasibility and safety of planned postoperative day 1 discharge (PPOD1) among patients who undergo XL at the department of gynecology oncology using a modified ERAS protocol including OSA and simplified discharge criteria.

## Methods

### Study design

**Figure 1:**
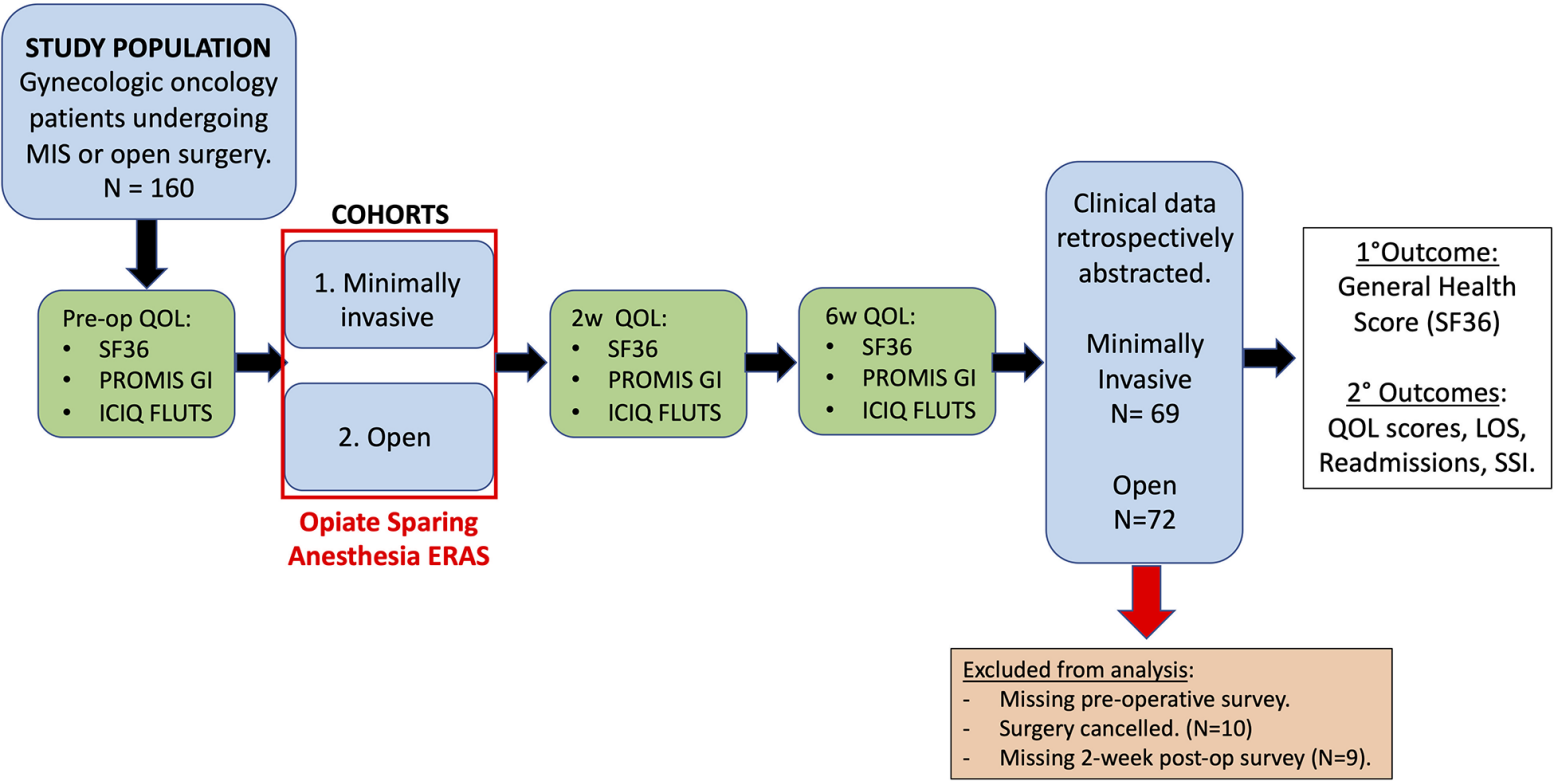
The study workflow. All patients scheduled surgery at our gynecologic oncology clinic between 1 May 2018 to 22 November 2019 were contacted for the study enrollment. A total of 160 patients were included after informed consents were obtained and patients were willing and able to complete our three quality-of-life surveys at each perioperative time point. Taking into account 20% drop out, accrual up to 80 patients in each cohort was allowed: laparotomy and minimally invasive approach. Patients were followed at 2 weeks and 6 weeks post-surgery. SF36, PROMIS GI, and ICIQ-FLUTS were assessed at each time point. Totally 141 patients were included for the final analysis. Clinical data of the 141 patients were retrospectively abstracted. SSI, surgical site infection.

This is a single-arm prospective study aimed to evaluate the feasibility and safety of modified ERAS protocol in patients undergoing surgery at a large quaternary care facility. The study was approved by the University of South Florida Institutional Review Board (IRB #00033144, approved on 26 February 2018). All patients who were presented to the gynecologic oncology clinic at the large quaternary care facility and scheduled for surgery between 1 May 2018 and 22 November 2019 were contacted for study enrollment if they met the study criteria. Figure 1 summarizes the study workflow. A total of 160 patients agreed to enroll in this study. The surgical approach was determined at the discretion of the physician. There were two cohorts, namely, (1) XL and (2) minimally invasive surgery (MIS). Given the established safety and efficacy of ERAS in MIS, this cohort served was considered as control (1). The primary objective was to evaluate the feasibility and safety of planned early discharge following laparotomy in gynecologic oncology with ERAS protocol including OSA and modified discharge criteria. Based on previous studies and our experiences, we anticipated low rates of complications and readmission. The primary outcome was SF36 general health score at the 2-week postoperative time point. This study was accrued to adequately power a quality of life (QoL) comparison between our target population undergoing XL and a control group of patients undergoing MIS. The secondary outcomes were also measured such as the rate of successful PPOD1, the rate of complications, the rate of unplanned readmissions, the amount of postoperative opioid use, the National Institutes of Health Patient-Reported Outcomes Measurement Information System Gastrointestinal (PROMIS GI) and the International Consultation on Incontinence Questionnaire for Female Lower Urinary Tract Symptoms (ICIQ-FLUTS) (6–9).

### Patient selection

All surgical candidates were considered eligible for this study if they were ≥18 years of age, were able to provide written informed consent, and indicated an ability and willingness to complete QoL surveys in English regardless of indications. The aim was to recruit 80 patients for each arm of the study. However, the patients who canceled surgeries or failed to complete surveys at a minimum of two time points were excluded from the analysis. All denoted clinical outcomes were recorded.

### Interventions

#### Modified ERAS protocol

The modified ERAS protocol is summarized in Table 1. The two main modifications we made to the ERAS criteria are the implementation of OSA and the simplification of the discharge criteria by redefining the return of bowel function (1). As described in the introduction, the current ERAS guidelines suggest using short-acting anesthetic agents like remifentanil for faster patient awakening, but perioperative opioids can hinder recovery due to various side effects (1). OSA is an alternative approach that uses a combination of non-opioid agents to achieve the same anesthetic effects with limited opioid use (5). Return of bowel function is redefined as the tolerance of a patient to a solid diet without vomiting, abdominal distension, or pain, regardless of the presence of flatus or passage of feces. Its rationale is discussed below. The rest of the protocol largely aligns with existing consensus guidelines. The patients received comprehensive preoperative counseling about postoperative expectations, including a review of the multimodal pain management plan such as acetaminophen, ketorolac or ibuprofen, lidocaine patch, gabapentin, abdominal binder, tramadol, and expected LOS following surgery, as described in Table 1. No components were modified for the study at any point—before, during, or after the enrollment.

**Table 1:** Components of modified enhanced recovery after surgery (ERAS) program.

Preoperative
Education and counseling: verbal and written information on postoperative expectations^a^
Preoperative optimization (e.g., address smoking, alcohol use, anemia, and diabetes)
Antimicrobial prophylaxis and skin preparation consistent with SCIP guidelines
Pre-treatment pain control and nausea (acetaminophen, gabapentin, scopolamine)
Regional anesthesia for open cases (transversus abdominis plane block or epidural)
Bowel preparation with magnesium citrate or GoLYTELY at 4 pm 1 day prior to surgery^b^
Clear liquids 2–4 h prior to surgery
Intraoperative
Short-acting anesthetic agents allowing for rapid awakening (e.g., propofol and sevoflurane)
Opioid-sparing anesthesia with limited use of long-acting opioids
Maintain tidal volume 5–7 ml/kg (usually <500 ml) and PEEP at 4–6 cm H_2_O
Multimodal antiemetics (ondansetron, metoclopramide, scopolamine)
Maintain intraoperative temperature above 36°C
Perioperative fluid management: maintain euvolemia
Postoperative
Multimodal approach to postoperative nausea and vomiting with >2 antiemetics
VTE prophylaxis: with extended prophylaxis for laparotomy for pelvic malignancy
Termination of IV fluids within 24 h after surgery
Regular diet within the first 24 h of surgery
Multi-agent bowel regimen of docusate oral or suppository and polyethylene glycol 3350
Postoperative glucose control <180
Multimodal analgesia^c^
Urinary catheter removed within 24 h of surgery
Early mobilization within 24 h of surgery
Postoperative day 0 discharge minimally invasive survery^d^
Postoperative day 1 discharge open surgery^d^
Discharge regimen: tramadol × 3 days, ibuprofen × 10 days, docusate × 30 days^d^
Simplified discharge criteria
Stable vital signs
Appropriate and stable postoperative hemoglobin or hematocrit
Adequate urine output with normal or stable kidney function
Ability to tolerate a solid diet without vomiting, abdominal distension, or pain
Ability to ambulate or move sufficiently enough to manage at current home environment
Ability to tolerate pain with a multimodal pain regimen
Voiding spontaneously unless a Foley catheter is clinically indicated
No social barrier for postoperative recovery after discharge

SCIP, Surgical Care Improvement Project; PEEP, positive end-expiratory pressure; VTE, venous thromboembolism.

^a^ Postoperative expectations include information on the procedure, pain, and length of stay.

^b^ GoLYTELY [polyethylene glycol 3350 227.1 g, sodium sulfate (anhydrous) 21.5 g, sodium bicarbonate 6.36 g, sodium chloride 5.53 g, potassium chloride 2.82 g]—patients were instructed to drink a total of up to 1 gal at a rate of 240 ml (8 oz.) every 10 min until 1 gal is consumed or the rectal effluent is clear. Magnesium citrate—patients were instructed to drink one bottle with four glasses of water at 4 pm on 1 day prior to surgery. Patients who underwent laparoscopic surgery at our department did not have bowel preparation.

^c^ Including acetaminophen, ketorolac or ibuprofen, lidocaine patch, gabapentin, abdominal binder, and tramadol.

^d^ If no contraindication.

#### Opioid-sparing anesthesia protocol

All study participants underwent general anesthesia using an opioid-sparing regimen, which was collaboratively developed with the anesthesiology team of our institution (5, 10). This regimen consists of propofol (0.2–0.4 μg/kg/h), ketamine (2–5 mg/kg bolus), magnesium [2 g intravenous (IV) bolus], and lidocaine (0.5–2 mg/kg/h). Ketamine and magnesium bolus were administered at the time of induction. A dexmedetomidine hydrochloride infusion (0.2–0.4 μg/kg/h) could be added at the discretion of the anesthesia provider if needed for additional intraoperative sedation. If the anesthesiology team deems the utilization of opioids imperative during the induction or surgical procedures, such administration is permitted and subject to their professional discretion. No extra monitor device was used outside of the routine monitoring system for general anesthesia. Bilateral transversus abdominis plane (TAP) blocks were routinely performed, using 30–40 cc of 0.25% plain ropivacaine, bilaterally, based on a weight-based algorithm. This was performed either preoperatively under ultrasound guidance by anesthesiologists or intraoperatively by a gynecologic oncology provider using O’Donnell's described method, depending on the perioperative logistics (11, 12).

#### The rationale of simplified discharge criteria

Our standardized hospital discharge criteria after surgery are summarized in Table 1. The systematic review of hospital discharge criteria following colorectal surgery reported that among the literature that cited return of bowel function, 46% defined “passage of stool,” 3% defined “passage of flatus and stool,” 19% defined “passage of flatus,” 15% defined passage of flatus or stool as return of bowel function (13). Although strict adherence to exhaustive criteria can be beneficial, it can also contribute to unnecessary discharge delays in otherwise healthy patients. This is particularly concerning given the complications associated with extended hospital stays and the challenges in healthcare systems including limited bed availability. Most surgeons acknowledge the criteria they refer to assess the readiness of the patients for discharge often lack evidence.

Emmanuel et al. (4) reported that redefining the return of bowel function to include the tolerance of a solid diet without vomiting, abdominal distension, or pain, regardless of the presence of flatus or passage of feces, enabled patients who underwent colorectal cancer resection to be safely discharged within 72 h after surgery without increasing the rate of complications. We hypothesized that we could safely implement the definition of return of bowel function by Emmanuel et al. to our gynecology oncology patients who underwent XL.

### Data collection

Data were extracted from our electronic database, such as patient demographics, comorbidities, preoperative body mass index (BMI), Eastern Cooperative Oncology Group (ECOG) functional status, tobacco use, preoperative hemoglobin value, and prior history of abdominal surgery or radiation (14). The clinical indication for surgery was obtained directly from surgery scheduling documentation (Table 2). The highest pain scores reported in nursing documentation within the considered timeframe were extracted. Postoperative day (POD) and LOS were quantified by the number of midnights spent in the hospital after surgery. Opioid use is reported in morphine milligram equivalents (MME), widely accepted clinical conversion standards. All anesthesiology records and medication reconciliation within electronic medical records were reviewed to ensure completeness in opioid administration tracking.

**Table 2:** Baseline subject demographics.

	All (*N* = 141)	MIS (*N* = 69)	XL (*N* = 72)	*p*-value
Age (years)	56.8 (13.4)	57.1 (11.5)	56.5 (15.0)	0.81
Race (ethnicity)				
Caucasian	104 (73.8)	54 (78.3)	50 (69.4)	0.43
Black	17 (12.1)	6 (8.7)	11 (15.3)	
Hispanic	13 (9.2)	7 (10.1)	6 (8.3)	
Other	5 (3.5)	2 (2.9)	3 (4.2)	
Unknown	2 (1.4)	0 (0)	2 (2.8)	
Body mass index	32.6 (9.2)	34.6 (8.9)	30.7 (9.1)	0.01
ECOG functional status				0.74
0	129 (91.5)	65 (94.2)	64 (88.9)	
1	10 (7.1)	4 (5.8)	6 (8.3	
2	1 (0.7)	0 (0)	1 (1.4)	
Medical comorbidities				
Hypertension	55 (39)	22 (31.9)	33 (45.8)	0.09
Cardiac disease	13 (9.2)	5 (7.2)	8 (11.1)	0.43
Diabetes	21 (14.9)	10 (14.5)	11 (15.3)	0.90
Psychiatric diagnosis	28 (19.9)	9 (13.0)	19 (26.4)	0.05
Total no. of comorbidities	2 (0–12)	2 (0–12)	2.5 (0–9)	0.08
Tobacco use				0.21
Never smoker	71 (50.4)	40 (58)	31 (43.1)	
Former smoker	51 (36.2)	21 (30.4)	30 (41.7)	
Current smoker	19 (13.5)	8 (11.6)	11 (15.3)	
Pre-op Hgb (g/dl)	12.9 (1.4)	13.1 (1.2)	12.6 (1.6)	0.03
Pre-op albumin (g/dl)	3.8 (0.4)	3.9 (0.3)	3.8 (0.5)	0.07
Prior radiation	5 (3.5)	3 (4.3)	2 (2.8)	0.68
Prior abdominal surgery	92 (65.2)	40 (58.0)	52 (72.2)	0.09
Indication for surgery				<0.001
Endometrial CA/EIN	50 (35.5)	37 (53.6)	13 (18.1)	
Ovarian ca	7 (5.0)	0 (0)	7 (9.7)	
Cervical ca	4 (2.8)	3 (4.3)	1 (1.4)	
Cervical dysplasia	10 (7.1)	7 (10.1)	3 (4.2)	
Adnexal mass	47 (33.3)	9 (13.0)	38 (52.8)	
Postmenopausal bleeding	8 (5.7)	6 (8.7)	2 (2.8)	
Risk-reducing surgery	8 (5.7)	7 (10.1)	1 (1.4)	
Leiomyoma/pelvic pain	7 (5.0)	0 (0)	7 (8.3)	

Pre-op, preoperative; Hgb, hemoglobin; ca, cancer; EIN, endometrial intraepithelial neoplasia.

Data presented as mean (standard deviation), median (range), and *n* (%) as indicated. Numbers may not add to the total due to missing values.

Health-related QoL was assessed using the SF36, an easily accessible patient-friendly survey that has been validated across diverse surgical populations (7). This instrument consists of 36 questions designed to evaluate eight domains of patient-reported health-related QoL including physical functioning (10 items), role limitations due to physical health (four items), role limitations due to emotional health (four items), bodily pain (two items), energy/fatigue (four items), mental health (five items), social functioning (two items), general health perceptions (five items), and one item assessing perceptions of health quality change over time (6). Possible scores for each question/item range from 0 to 100 with a higher score representing positive QoL. Domain-specific scores are then calculated by the total values of each item based on response.

Given the potential risk of GI complications following abdominal surgery and opioid-related GI side effects, we incorporated the PROMIS GI survey to assess the GI symptoms (8). The PROMIS GI constipation scale assesses the frequency and intensity of incomplete stool evacuation, need for assistance with stool evacuation, rectal pain, straining, and hard stools. The PROMIS GI nausea and vomiting scale assesses the frequency of vomiting, nausea, and poor appetite. Scoring is assigned based on a predetermined *T* score associated with the sum of the survey answers, with a higher score representing more significant symptomatology.

Finally, we identified urinary symptoms, consequential to both gynecologic surgery and opioid use, as a significant factor for QoL. The ICIQ-FLUTS was used to evaluate lower urinary tract symptoms (LUTS) and their impact on QoL (9). ICIQ-FLUTS is derived from the previously validated Bristol female lower urinary tract symptom questionnaire (9). Symptom questions are grouped into whether they are related to bladder filling, voiding, or incontinence. Answers to each question correlate with a numeric score, i.e., a filling score (*F* score), voiding score (*V* score), and incontinence score (*I* score), in which higher summed scores indicate worse symptoms.

### Outcome measures and statistical analysis

The primary outcome of this study is an SF36 general health score at the 2-week postoperative time point. This provides a comprehensive view of QoL in the early recovery period. Secondary outcomes include additional SF36 QoL domains, GI and urinary symptom-related QoL, surgical site infection rate, LOS, postoperative patient phone calls, and readmission rates. As described in the study design, an MIS group as a control group represents perioperative QoL as a comparison to our cohort of interest undergoing XL. The study was then powered to assess the QoL performance of the XL group undergoing ERAS with OSA and POD1 discharge compared to the MIS group following the same ERAS protocol listed in Table 1.

Our null hypothesis posited that there were no significant differences in the 2-week postoperative general health score between the MIS and XL groups. To achieve 80% statistical power to reject the null hypothesis, we calculated that 80 patients should be enrolled in each cohort, accounting for an estimated 20 dropout rate to accrue at least 64 subjects (80%) with available primary outcome data in each cohort.

Baseline characteristics were compared using Fisher's exact test or chi-square test. QoL and clinical outcomes were compared between cohorts using the Student's *t*-test and non-parametric Mann–Whitney two-sample test. The subjects were excluded from analysis if their surgery was canceled or if they failed to complete the SF36 questionnaire at the preoperative time point and at least one of two possible postoperative time points.

## Results

A total of 160 patients were enrolled in this study from 1 May 2018 to 22 November 2019. The QoL surveys took approximately 10–15 min to complete. Slow accrual was mainly due to unwillingness to dedicate those surveys. Two-week survey responses were from 141 subjects. Ten subjects (four from the planned XL group and six from the planned MIS group) were subsequently excluded from analyses because they did not undergo surgery. An additional nine subjects (two from the XL group and seven from the MIS group) were excluded because they did not complete the QoL surveys at the 2-week postoperative visit.

### Baseline subject demographics

The demographics of the 141 subjects are summarized in Table 2. A total of 69 patients underwent MIS, whereas 72 patients underwent XL. Compared to the XL group, the MIS group had a higher median BMI (34.6 vs. 30.7; *p* = 0.01) and a lower prevalence of pre-existing mental health issues (13% vs. 26.4%; *p* = 0.05). The most commonly reported mental health issue was anxiety. The majority of preoperative diagnoses were endometrial cancer or endometrial hyperplasia (*N* = 37, 53.6%) in the MIS group, whereas the majority of XL cases were performed for an adnexal mass (*N* = 38, 52.8%).

### Surgical characteristics

Table 3 presents the surgical details. The MIS group had a higher rate of patients who underwent hysterectomy (98.6% MIS vs. 75.0 XL, *p* = <0.001), and the XL group had a lower rate of patients who underwent para-aortic lymphadenectomy (5.8% MIS vs. 22.2% XL, *p* = 0.005), omentectomy (5.8% MIS vs. 31.9% XL, *p* = 0.005), or bowel resection (0% MIS vs. 6.9% XL, *p* = 0.060). Total surgical time (96.8 min MIS vs. 114.2 min XL, *p* = 0.06) and estimated blood loss (33.4 ml MIS vs. 74.5 ml XL, *p* = 0.015) were also statistically higher in the XL group. The MIS group was more frequently diagnosed with endometrial cancer (34.8% MIS vs. 18.1% XL, *p* = 0.035) and endometrial hyperplasia (20.3% MIS vs. 0% XL, *p* = <0.001). In contrast, those undergoing XL were more often diagnosed with ovarian cancer or borderline ovarian tumor (1.4% MIS vs. 18.1% XL, *p* = 0.001). The XL group had a higher rate of patients who underwent surgery for gynecologic malignancy [XL group 61 (84.7%) vs. MIS group 21 (29.2%), *p* < 0.001]. In the XL group, endometrial cancer or endometrial intraepithelial neoplasia and ovarian cancer or borderline tumor are the most common indications among patients with suspected malignancy (18.1% and 18.1, respectively).

**Table 3:** Surgical characteristics.

	All (*N* = 141)	MIS (*N* = 69)	XL (*N* = 72)	*p*-value
Hysterectomy	122 (86.5)	68 (98.6)	54 (75.0)	<0.001
Pelvic LND	42 (29.8)	20 (29.0)	22 (30.6)	0.840
Para-aortic LND	20 (14.2)	4 (5.8)	16 (22.2)	0.005
Omentectomy	27 (19.1)	4 (5.8)	23 (31.9)	<0.001
Bowel resection	5 (3.5)	0 (0)	5 (6.9)	0.060
Total surgical time (min)	105.7 (37.8)	96.8 (32.1)	114.2 (41.0)	0.006
Estimated blood loss (ml)	25 (2–800)	20 (5–500)	50 (2–800)	<0.001
Pathologic diagnosis				<0.001
Endometrial Ca	37 (26.2)	24 (34.8)	13 (18.1)	
Ovarian Ca/borderline tumor	14 (9.9)	1 (1.4)	13 (18.1)	
Cervical Ca	4 (2.8)	3 (4.3)	1 (1.4)	
Endometrial hyperplasia	14 (9.9)	14 (20.3)	0 (0)	
Benign ovarian	40 (28.4)	15 (21.7)	25 (34.7)	
Benign uterine	17 (12.1)	5 (7.2)	12 (16.7)	
Cervical dysplasia	10 (7.1)	7 (10.1)	3 (4.2)	
Benign gastrointestinal	3 (2.1)	0 (0)	3 (4.2)	
Malignant non-gyn primary	2 (1.4)	0 (0)	2 (2.8)	
Stage of malignancy				0.001
1	36 (25.5)	23 (33.3)	13 (18.1)	
2	4 (2.8)	0 (0)	4 (5.6)	
3	9 (6.4)	2 (2.9)	7 (9.7)	
4	4 (2.8)	0 (0)	4 (5.6)	
N/A	88 (62.4)	44 (63.8)	44 (61.1)	

LND, lymphadenectomy; Ca, cancer; N/A, not applicable.

Data presented as *n* (%), mean (standard deviation), or median (range) as appropriate.

### Perioperative clinical outcomes

The postoperative outcomes for the subjects enrolled in this study are summarized in Table 4. Administration of opioids outside of OSA protocol during surgery and postanesthesia care unit (PACU) was at the discretion of the anesthesiologists, and opioid administration after PACU was at the discretion of the surgeons. More patients in the XL group received opioids (fentanyl) during the general anesthesia induction compared to the MIS group (1.4% vs. 36.1%, *p* = <0.01). The XL group required more opioids intraoperatively (7.1 ± 9.2 MME) compared to the MIS group (3.9 ± 6.9 MME, *p* = 0.02). There was no difference in the amount of opioids needed at the immediate postoperative phase (PACU), the rest of the POD 0, and POD 1. There was no difference in the highest reported pain score at PACU and POD1. In the XL group, 53 patients (73.6%) met the discharge criteria and were successfully discharged home on POD1. Within the 6-week follow-up period, we found no significant differences between the two groups regarding surgical site infection rates (MIS 0% vs. XL 2.8%, *p* = 0.24), median number of patient phone calls to the office [MIS 0 (0–3) vs. XL 0 (0–4), *p* = 0.41], or readmission rates less than 30 days from surgery (MIS 1.4% vs. XL 4.2%, *p* = 0.62). Notably, there were no patient deaths identified during the follow-up period.

**Table 4:** Perioperative clinical measures.

	MIS (*N* = 69)	XL (*N* = 72)	*p*-value
Highest reported pain score			
PACU	4.14 ± 3.38	4.2 ± 3.4	0.52
POD 1	4.6 ± 2.4	4.5 ± 2.6	0.58
Narcotics administered (MME)			
Intra op	3.9 ± 6.9	7.1 ± 9.2	0.02
PACU	5.5 ± 6.5	7.4 ± 7.4	0.11
POD 0	0.1 ± 0.14	1.4 ± 3.6	0.38
POD 1	0.31 ± 1.95^a^	2.3 ± 12.5	0.38
Regular diet tolerated (POD#)	0 (0–1)	0 (0–4)	<0.001
Foley catheter removed (POD)	0 (0–1)	1 (0–19)	<0.001
Length of hospital stay (days)	0 (0–2)	1 (0–11)	<0.001
Surgical site infection (No. subjects)	0 (0)	2 (2.8)	0.24
Post-discharge phone calls (# calls)	0 (0–3)	0 (0–4)	0.41
Readmissions <30 days	1 (1.4)	3 (4.2)	0.62

^a^ One subject with a prior history of opioid use disorder was excluded.

### Quality of life outcomes

QoL outcomes were assessed for two cohorts, namely, the cohort of interest undergoing XL and a control cohort undergoing MIS. All participants in both cohorts adhered to the ERAS protocol as described in Table 1. As demonstrated in Table 5, the cohorts were comparable for baseline/preoperative QoL scores in all measured domains of SF36, ICIQ-FLUTs, and PROMIS GI scores.

**Table 5:** Patient-reported QoL outcomes.

	Preoperative				2-week postoperative				6-week postoperative			
	*N*	MIS	*N*	XL	*N*	MIS	*N*	XL	*N*	MIS	*N*	XL
SF36												
Physical functioning	65	72.1 (27.6)*	71	68.6 (26.9)*	63	55.9 (28.8)*^,^**	71	39.9 (28.5)*^,^**	59	66.9 (23.5)	57	61.5 (30.0)*
Role: physical health	69	58.7 (43.1)*	72	59.0 (43.7)*	67	26.1 (38.5)*^,^**	72	13.5 (27.2)*^,^**	61	51.6 (40.5)**	59	31.4 (38.2)*^,^**
Role: emotional problems	68	68.6 (43.8)*	72	70.4 (41)	68	69.1 (40.5)	71	59.2 (46.2)	61	80.9 (35.2)*	58	67.2 (43.1)
Energy/fatigue	68	50.7 (23.5)	72	48.2 (23.1)*	66	45.2 (20.8)	72	41.9 (19.9)*	61	54.2 (21.9)	58	51.0 (21.7)
Emotional wellbeing	68	72.3 (16.6)*	72	69.9 (20.7)*	68	73.6 (19.3)	71	71.8 (18.3)	62	77.9 (15.9)*	60	76.9 (17.8)*
Social functioning	67	74.0 (27.9)*	71	71.4 (25.7)*	69	62.0 (28.2)*^,^**	71	49.5 (27.5)*^,^**	61	75.4 (26.5)	60	65.6 (29.4)*
Pain	67	64.3 (27.1)*	72	58.7 (29.2)*	67	50.1 (23.9)*^,^**	71	39.7 (25.4)*^,^**	61	64.3 (24.5)	59	63.5 (23.8)
General health	68	65.0 (20.8)	71	64.0 (22.2)	67	65.6 (22.1)	72	66.3 (21.6)	60	70.0 (19.6)	57	68.2 (21.7)
Health change	68	46.3 (26.0)*	72	42.7 (23.1)*	69	56.2 (28.6)*	72	50.7 (24.8)*	61	65.6 (28.9)*	61	58.3 (29.5)*
ICIQ-FLUTS												
FLUTS *F* score	65	4.0 (2.9)	72	4.9 (3.3)*	63	4.0 (2.8)	71	4.5 (3.1)	59	3.9 (2.5)	58	3.7 (2.8)*
FLUTS *V* score	67	1.3 (1.7)*^,^**	71	1.9 (2.5)**	68	2.0 (2.1)*	71	2.0 (2.3)	62	1.5 (1.9)	59	1.3 (1.7)
FLUTS *I* score	67	4.5 (4.2)*	71	4.7 (4.0)*	66	3.2 (3.4)*	71	3.5 (3.7)*	61	4.0 (4.1)	58	4.1 (4.4)
Overall FLUTS score	63	9.6 (6.6)	71	11.5 (8.1)*	63	9.0 (6.6)	70	9.8 (7.1)*	59	9.4 (6.4)	57	9.2 (7.7)
PROMIS GI												
Constipation	58	13.3 (6.5)	59	14.2 (9.0)	62	14.1 (7.3)	62	13.7 (7.7)	61	12.8 (6.8)	49	14.9 (9.4)
Nausea/vomiting	59	5.6 (2.8)	60	6.1 (3.7)	55	5.5 (3.1)	60	6.7 (3.8)	47	5.1 (2.7)	53	5.2 (2.6)

Bold indicate statistically significant factors. Data presented as mean (standard deviation).

A higher score for SF36 denotes improvement in symptoms. A higher score for ICIQ-FLUTS and PROMIS GI denotes worsening symptoms.

*p*-values for between groups were obtained using the Student's *t*-test.

*p*-values for within groups were obtained using the paired *t*-test.

Preoperative vs. week 2 or week 6.

* *p* < 0.05. Minimally invasive vs. open.

** *p* < 0.05.

There was no difference between the two cohorts in general health scores.

In terms of secondary outcome, both MIS and XL groups had significantly lower SF36 scores in the areas of physical functioning, role in physical health, social functioning, and pain, as compared to their respective baseline scores. The decline was more pronounced in the XL group compared to the MIS group (*p* < 0.05). Among the four domains, all except physical health recovered by the 6-week time point.

In addition, the XL group exhibited a significant decrease in the domain of energy/fatigue at 2 weeks postoperatively but recovered by the 6-week time point.

With regard to urinary symptoms at this time point, patients in the MIS group reported more voiding symptoms. Both MIS and XL groups reported improved incontinence symptoms at the 2-week postoperative time point compared to baseline.

At the 2-week postoperative time point, both groups did not experience worsening GI symptoms, as measured by PROMIS GI, and no significant differences were found.

## Discussion

In the late 1990s, Dr. Henrik Kehlet (15) theorized that attenuating the surgical stress response through perioperative interventions could positively affect postoperative morbidity and expedite surgical recovery. Over time, these ideas have evolved to form the foundation of the ERAS protocol aimed at optimizing postoperative recovery. However, there is still variability in individual components of ERAS programs, such as anesthetic technique and timing of postoperative discharge. A consensus has yet to be reached on the safest approach that yields the most optimal postoperative recovery (3, 16, 17).

As we have previously described, our simplified discharge criteria consider patients to have returned to normal bowel function once they tolerated a solid diet regardless of passing flatus or feces. This aligns with the universal discharge criteria already in place for patients undergoing MIS. The implementation of these criteria in the XL group did not increase the complication or readmission rates and the PROMIS GI constipation score and nausea and vomiting score compared to the MIS group in our study. These findings support the safety of our simplified discharge criteria.

The current ERAS guidelines in gynecology oncology do not address intraoperative opioid administration, which can be a significant factor. Our data demonstrate that incorporating an OSA regimen into an existing ERAS program is safe and can be implemented without exacerbating perioperative pain for patients undergoing major gynecologic surgery via either XL or MIS. The use of opioids outside of OSA protocol at the discretion of anesthesiologists or surgeons was found in the analysis; however, the amount of opioid use was lower than that previously reported in the literature (5). The completion of surgery without a major change in the anesthesiologic approach and the acceptable amount of opioids used at the perioperative phase suggest the feasibility of this anesthetic approach.

QoL including physical, psychological, social, and functional components was used as a measure to characterize subjective recovery after surgery and patient tolerability of the presented protocol (3, 8). Our study focused on patients undergoing gynecologic oncology surgery under a specialized ERAS protocol, comparing outcomes to a control MIS group. The QoL data show no difference in general health QoL scores at any measured time point between cohorts. We also demonstrated a positive perceived health change as early as 2 weeks in the XL group which increased even further by 6 weeks and did not statistically differ from the MIS scores. These findings suggest well-performing postoperative recovery after XL following this specialized ERAS protocol compared to a control MIS group.

In a further evaluation of additional QoL domains, four out of the nine domains favor the MIS group at 2 weeks postoperatively. Although general health scores remained stable throughout the recovery periods, recovery in the XL group continues to lag behind MIS, particularly in the early 2-week postoperative period. However, by the 6-week postoperative time point, the two cohorts had comparable scores for all QoL domains, except for “role: physical health.” When compared to prior studies like LAP2 and LACE, which showed superior QoL in MIS, our findings suggest that our ERAS interventions may significantly improve postoperative recovery, particularly for patients undergoing XL (18, 19).

Although our results are encouraging that OSA and early hospital discharge following XL may hasten surgical recovery, further prospective studies in a larger population are needed to investigate the unique contributions of these components to an ERAS protocol for establishing an optimal protocol for surgical recovery.

The strengths of this study include the use of validated, patient-reported QoL measures at predetermined time points, and the diverse study population representing the heterogeneous surgical procedures performed in the gynecologic oncology department. This study also faces several limitations including a non-randomized design, the absence of a non-OSA comparison group, and a limited time frame for postoperative assessment. The lack of randomization between surgical cohorts is reflected in the differences in baseline surgical characteristics, pathology, and surgical procedures performed between cohorts. Although a recent study suggests spinal anesthesia could be an alternative option in selected laparoscopic gynecologic cases to reduce postoperative analgesic usage, our study only focused on the context of general anesthesia (20). It is important to consider these factors when interpreting differences in QoL between two surgical cohorts. Despite the more extensive surgery and malignant pathology in the XL group, little difference was noted in QoL compared to MIS at 6 weeks postoperatively. We chose a 6-week postoperative time point as the maximum follow-up duration for several reasons. First, most patients with benign conditions typically do not require further follow-up with our department beyond this period. Therefore, asking them to return solely for research purposes would place an unnecessary financial and physical burden on them. Second, evaluations after a 6-week postoperative time point could be impacted by adjuvant therapy among patients with malignancies. It is reported that patients who were admitted due to acute respiratory infection still have decreased QoL 1 year after hospital discharge. Future studies are warranted to evaluate the long-term QoL following gynecology surgery, which potentially addresses invaluable factors impacting QoL (21). Our study had a relatively slow accrual due to the study population being limited to those willing to complete QoL surveys at perioperative visits which may have led to bias in patient selection.

Despite these limitations, our data demonstrated that PPOD1 is feasible for women undergoing gynecologic surgery without any apparent adverse outcomes using modified ERAS protocol including OSA and simplified discharge criteria PPOD1. This study successfully addresses the paucity of data on OSA and simplified discharge criteria in the field of gynecology oncology.

## Conclusions

PPOD1 is safe and feasible using a modified ERAS protocol including OSA and our defined discharge criteria for XL performed at the gynecologic oncology department without increasing the need for opioids, readmission rates compared to MIS, and patient-reported constipation and nausea/vomiting compared to the preoperative phase.

## Data Availability

The raw data supporting the conclusions of this article will be made available by the authors, without undue reservation.
